# The association between postoperative photon radiotherapy dose and disease control and salvage treatment in pediatric and adolescent ependymoma: a multi-institutional investigation

**DOI:** 10.1007/s11060-025-04975-5

**Published:** 2025-02-25

**Authors:** Kevin X. Liu, Mia Salans, Teresa P. Easwaran, Christina Phuong, Kee Kiat Yeo, Hesham Elhalawani, Paul J. Catalano, Kathryn Dusenbery, Karen J. Marcus, Stephanie A. Terezakis, Daphne A. Haas-Kogan, Steve E. Braunstein

**Affiliations:** 1 Department of Radiation Oncology, Brigham and Women’s Hospital, Dana-Farber Cancer Institute, Boston Children’s Hospital, Harvard Medical School, Boston, MA USA; 2 Department of Radiation Oncology, University of California at San Francisco and UCSF Benioff Children’s Hospital, 1600 Divisadero St., Suite H1031, San Francisco, CA 94143-1708 USA; 3 Department of Radiation Oncology, University of Minnesota, Minneapolis, MN USA; 4 Department of Pediatrics, Dana-Farber/Boston Children’s Cancer and Blood Disorders Center, Boston, MA USA; 5 Department of Biostatistics, Department of Biostatistics and Computational Biology, Harvard T.H. Chan School of Public Health, Dana-Farber Cancer Institute, Boston, MA USA

**Keywords:** Intracranial ependymoma, Adjuvant radiation, Radiation dose, Relapsed ependymoma

## Abstract

**Purpose:**

We characterized the association between photon radiation dose (< 59.4 versus ≥ 59.4 Gy) and outcomes in intracranial ependymoma. We also examined factors associated with survival after relapse.

**Methods:**

This multi-institutional retrospective study included patients age ≤ 21 years who received postoperative definitive-intent photon radiotherapy for posterior fossa ependymoma between 1997 and 2021. Clinical characteristics were obtained from medical records. Five-year overall (OS) and progression-free (PFS) survival were estimated using the Kaplan-Meier method. Factors associated with progression after radiotherapy, including dose < 59.4 versus ≥ 59.4 Gy, were analyzed using Fine and Gray’s proportional subhazards model. Factors associated with post-relapse survival were explored using the Cox proportional-hazards model.

**Results:**

We identified 45 patients meeting inclusion criteria; 48.9% received ≥ 59.4 Gy. There was no difference in 5-year OS or PFS between those who received < 59.4 Gy versus ≥ 59.4 (OS 49.0% vs. 82.9%, *p* = 0.11; PFS 36.4% vs. 63.9%, *p* = 0.08); however, there was a trend towards worse 5-year OS and PFS among patients with grade 2 ependymoma who received < 59.4 Gy (OS 48.8% vs. 88.9%, *p* = 0.06, PFS 40.0% vs. 83.1%, *p* = 0.08). Only age > 4 years at diagnosis (subdistribution hazard ratio [SHR]: 0.40, *p* = 0.03) was associated lower risk of progression. Following radiotherapy, 24 patients relapsed. Receipt of salvage systemic therapy was associated with worse post-relapse OS on multivariable analysis (HR = 2.84, *p* = 0.04).

**Conclusion:**

Underlying biological factors such as age and molecular subtype may hold greater prognostic significance than radiation dose in pediatric ependymoma. Regardless, recurrences are common and outcomes remain poor. Further research into optimal management of relapsed disease is critical.

**Supplementary Information:**

The online version contains supplementary material available at 10.1007/s11060-025-04975-5.

## Introduction

Ependymomas are rare central nervous system tumors that primarily occur in children younger than two years old [1]. Postoperative radiotherapy has been shown to improve local control and survival in ependymoma [2–4] and is therefore the standard of care in most patients over one year of age. Radiation planning must carefully balance the risks of local failure with injury to nearby critical structures, particularly the brainstem. Given the relative rarity of ependymoma, there are no randomized data exploring varying dose and fractionation regimens. Moreover, many studies of radiotherapy are confounded by selection of dose based on adverse prognostic features such as histologic grade. Current standard doses range from 54 Gy to 59.4 Gy, with ACNS0121 recommending 54 Gy for patients < 12–18 months old after gross total resection (GTR) and 59.4 Gy for all other patients [2]. Nevertheless, optimal radiotherapy dosing remains unclear. While dose escalation is appealing due to ependymomas’ tendency to recur locally [5], this must be balanced with long-term, radiation-induced central nervous system toxicities including cognitive and neuroendocrine dysfunction, hearing deficits, and developmental delay [6–8].

In this multi-institutional, retrospective cohort study, we sought to characterize outcomes after photon-based radiotherapy for intracranial ependymoma. Specifically, we explored survival in patients who received < 59.4 Gy versus ≥ 59.4 Gy, accounting for clinical factors including age, tumor grade and location, and extent of resection. We also examined factors associated with survival in a subgroup of patients with relapsed disease.

## Methods

This retrospective cohort study was approved by the Institutional Review Board at each participating institution. Patients with a pathologically confirmed diagnosis of localized, grade 2 or 3, posterior fossa ependymoma who received definitive photon radiotherapy between 1997 and 2021 were included. All patients were treated with 3-D conformal radiotherapy or intensity-modulated/volumetric modulated arc photon-based radiotherapy. Patients were excluded if they were > 21 years of age at diagnosis or had no documented institutional follow-up after radiotherapy. Electronic and paper medical records were reviewed and data on clinical characteristics, including age, sex, race/ethnicity, location of primary tumor, histologic grading of the tumor, and sites of metastatic disease were collected. We also collected treatment data, including extent of surgical resection (i.e., gross total resection [GTR] versus subtotal resection [STR]), number of surgeries, use of systemic therapy, radiotherapy dose and fractionation, and any treatments at the time of disease progression. Patients were classified as having received ≥ 59.4 Gy versus < 59.4 Gy based on the highest subvolume prescription dose in their radiation treatment plans. Upfront extent of resection and radiologic progression were documented by each institution’s clinical team and re-reviewed by the study authors. We also reviewed radiation plans and imaging at the time of progression to determine location of disease progression relative to the initial radiation field. Follow-up information, including date of last clinical follow-up, date of death, dates of local and metastatic progression, and dates of secondary malignancy, were also recorded.

STATA v15.0 (Stata Corp, College Station, TX) and R (R Core Team [9]) were used for all statistical analyses. The primary objective of this study was to characterize 5-year progression-free survival (PFS) and overall survival (OS) for this cohort. Secondary objectives were to characterize the 5-year cumulative incidence of local progression (CILP) of the entire cohort, describe the 5-year PFS, OS, and CILP stratified by histological grade, and determine whether baseline or treatment characteristics were associated with progression of disease. Additional exploratory analyses were performed to examine whether baseline or upfront treatment characteristics and disease characteristics at relapse were associated with post-relapse survival. Follow-up was defined from the completion of radiotherapy. PFS and OS were analyzed using the Kaplan-Meier method and compared with the Gehan-Breslow-Wilcoxon test to highlight early recurrences/deaths given that late relapses (> 5 years after treatment) can occur in ependymoma [10]. Cumulative incidence of any progression and CILP were analyzed using competing risks of death with Gray’s test. Fine and Gray’s proportional subhazards model was used for univariable and multivariable analyses of factors associated with progression using death as a competing risk. The Cox proportional hazards model was used for univariable analyses of factors associated with death for patients with relapse after radiotherapy. Radiation dose stratified by ≥ 59.4 Gy and < 59.4 Gy and variables that were significant on univariable analyses were included for multivariable analysis. Categorical variables were analyzed using Fisher exact tests and quantitative variables were analyzed using the Mann-Whitney test. P-values < 0.05 were considered statistically significant.

## Results

### Patient and treatment characteristics

We identified 45 patients meeting our inclusion criteria. Twenty-three (51.1%) patients received < 59.4 Gy and 22 (48.9%) received ≥ 59.4 Gy at the time of upfront radiotherapy. One patient received 49.2 Gy and was excluded. Four patients (88.9%) were treated with three-dimensional conformal radiotherapy and the remainder received intensity-modulated radiation therapy/volumetric modulated arc therapy. Table 1 summarizes the characteristics of the two groups stratified by radiation dose. The median prescribed dose in the < 59.4 Gy cohort was 54 Gy (53.1 Gy: *n* = 1, 54.0 Gy, *n* = 10, 55.8 Gy, *n* = 12) and the median prescribed dose in the ≥ 59.4 Gy cohort was 59.4 Gy (59.4 Gy: *n* = 21, 60.3 Gy: *n* = 1). The median ages at diagnosis were 2.6 years (range: 0.9–12.5) and 9.1 years (range: 0.9–19.2) for the < 59.4 Gy and ≥ 59.4 Gy cohorts, respectively (*p* = 0.01). Only nine (20.0%) patients in the entire cohort underwent STR and extent of resection was balanced between the two dose groups. We found that significantly more patients were ≤ 4 years of age at time of diagnosis (65.2% vs. 27.3%, *p* = 0.02) and significantly fewer patients had grade 3 histology (8.7% vs. 40.9%, *p* = 0.02) within the < 59.4 Gy cohort. In addition, significantly more patients received post-radiotherapy chemotherapy (31.8% vs. 0.0%, *p* = 0.004) in the ≥ 59.4 Gy cohort. This was due to many patients receiving post-radiation chemotherapy as per the ACNS0831 protocol [11] after the trial was opened [12]. This practice was discontinued after the abstract results were published showing no significant difference in survival.

**Table 1:** Clinical and treatment characteristics

	Entire Cohort *n* (%)	< 59.4 Gy *n* (%) (*n* = 23)	≥ 59.4 Gy *n* (%) (*n* = 22)	*p*-value
Sex				0.76
Female	15 (33.3%)	7 (30.4%)	8 (36.4%)	
Male	30 (66.7%)	16 (69.6%)	14 (63.6%)	
Race/Ethnicity				0.21
Non-Hispanic White	31 (68.9%)	18 (78.3%)	13 (59.1%)	
Other	14 (31.1%)	5 (21.7%)	9 (40.9%)	
Age at diagnosis				0.02*
≤4 years	21 (46.7%)	15 (65.2%)	6 (27.3%)	
>4 years	24 (53.3%)	8 (34.8%)	16 (72.7%)	
Hydrocephalus at Diagnosis				0.31
No	11 (24.4%)	4 (17.4%)	7 (31.8%)	
Yes	34 (75.6%)	19 (82.6%)	15 (68.2%)	
Histological Grade				0.02*
Grade 2	34 (75.6%)	21 (91.3%)	13 (59.1%)	
Grade 3	11 (24.4%)	2 (8.7%)	9 (40.9%)	
Pre-RT chemotherapy				1.00
No	36 (80.0%)	18 (78.3%)	18 (81.8%)	
Yes	9 (20.0%)	5 (21.7%)	4 (18.2%)	
Disease Progression before RT				0.19
No	39 (86.7%)	18 (78.3%)	21 (95.5%)	
Yes	6 (13.3%)	5 (21.7%)	1 (4.5%)	
Resection status before RT				1.00
GTR	36 (80.0%)	18 (78.3%)	18 (81.8%)	
STR	9 (20.0%)	5 (21.7%)	4 (18.2%)	
Number of surgeries before RT				0.17
1	34 (75.6%)	15 (65.2%)	19 (86.4%)	
2 or more	11 (24.4%)	8 (34.8%)	3 (13.6%)	
Post-RT chemotherapy				0.004*
No	38 (84.4%)	23 (100.0%)	15 (68.2%)	
Yes	7 (15.6%)	0 (0.0%)	7 (31.8%)	
Days after last surgery to RT				0.75
≤42 days	13 (28.9%)	6 (26.1%)	7 (31.8%)	
>42 days	32 (71.1%)	17 (73.9%)	15 (68.2%)	

*Indicates significant at *p* < 0.05

### Overall survival

**Fig. 1:**
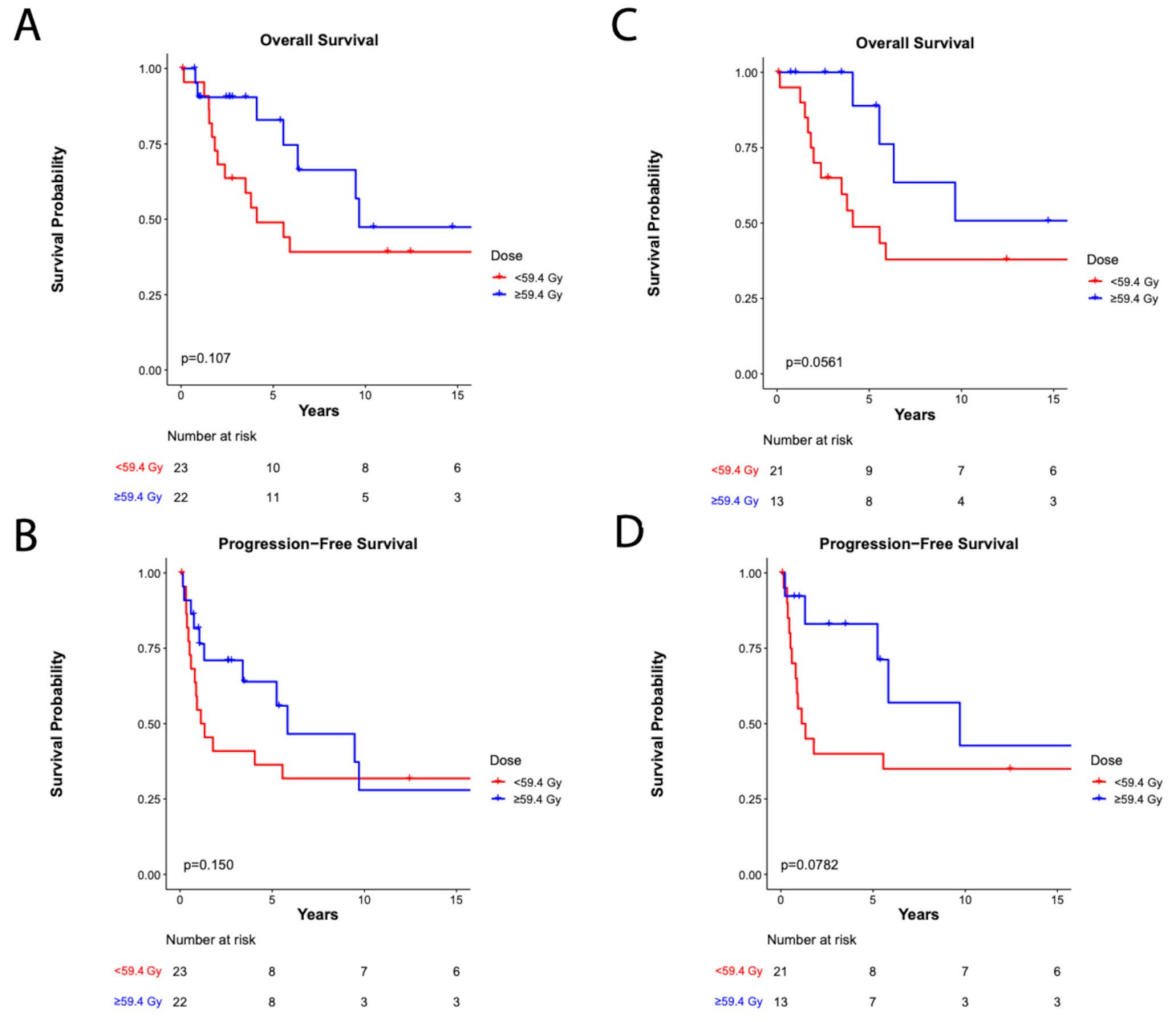
Survival outcomes. **(a)** Overall survival of the entire cohort (*p* = 0.11, Gehan-Breslow-Wilcoxon test). **(b)** Progression-free survival of the entire cohort (*p* = 0.15, Gehan-Breslow-Wilcoxon test). **(c)** Overall survival of patients with grade 2 ependymoma (*p* = 0.06, Gehan-Breslow-Wilcoxon test). **(d)** Progression-free survival of patients with grade 2 ependymoma (*p* = 0.08, Gehan-Breslow-Wilcoxon test)

The median follow-up times after radiation treatment were 3.8 years (range: 0.1–24.0) and 5.4 years (range: 0.7–24.0) for the < 59.4 Gy and ≥ 59.4 Gy cohorts, respectively (*p* = 0.76). Five-year OS was 63.9%, 49.0% and 82.9% for the entire cohort, the < 59.4 Gy cohort, and ≥ 59.4 Gy cohort, respectively (*p* = 0.11) and 5-year PFS was 46.3%, 36.4% and 63.9% for the entire cohort, the < 59.4 Gy cohort, and the ≥ 59.4 Gy cohort, respectively (*p* = 0.15) (Fig. 1a-b). There was no difference in PFS or OS between the ≤ 54 Gy, 55.8 Gy, and ≥ 59.4 Gy subgroups (*p* = 0.27) (Supplemental Figure S1). Given the imbalance of histologic grade between the two cohorts, we next examined outcomes among patients with grade 2 ependymoma. Among these patients, 5-year OS and 5-year PFS was 62.9% and 56.1%, respectively. Within the < 59.4 Gy and ≥ 59.4 Gy cohorts, 5-year OS and 5-year PFS was 48.8% and 88.9% (*p* = 0.06) and 40.0% and 83.1% (*p* = 0.08), respectively (Fig. 1c-d).

**Fig. 2:**
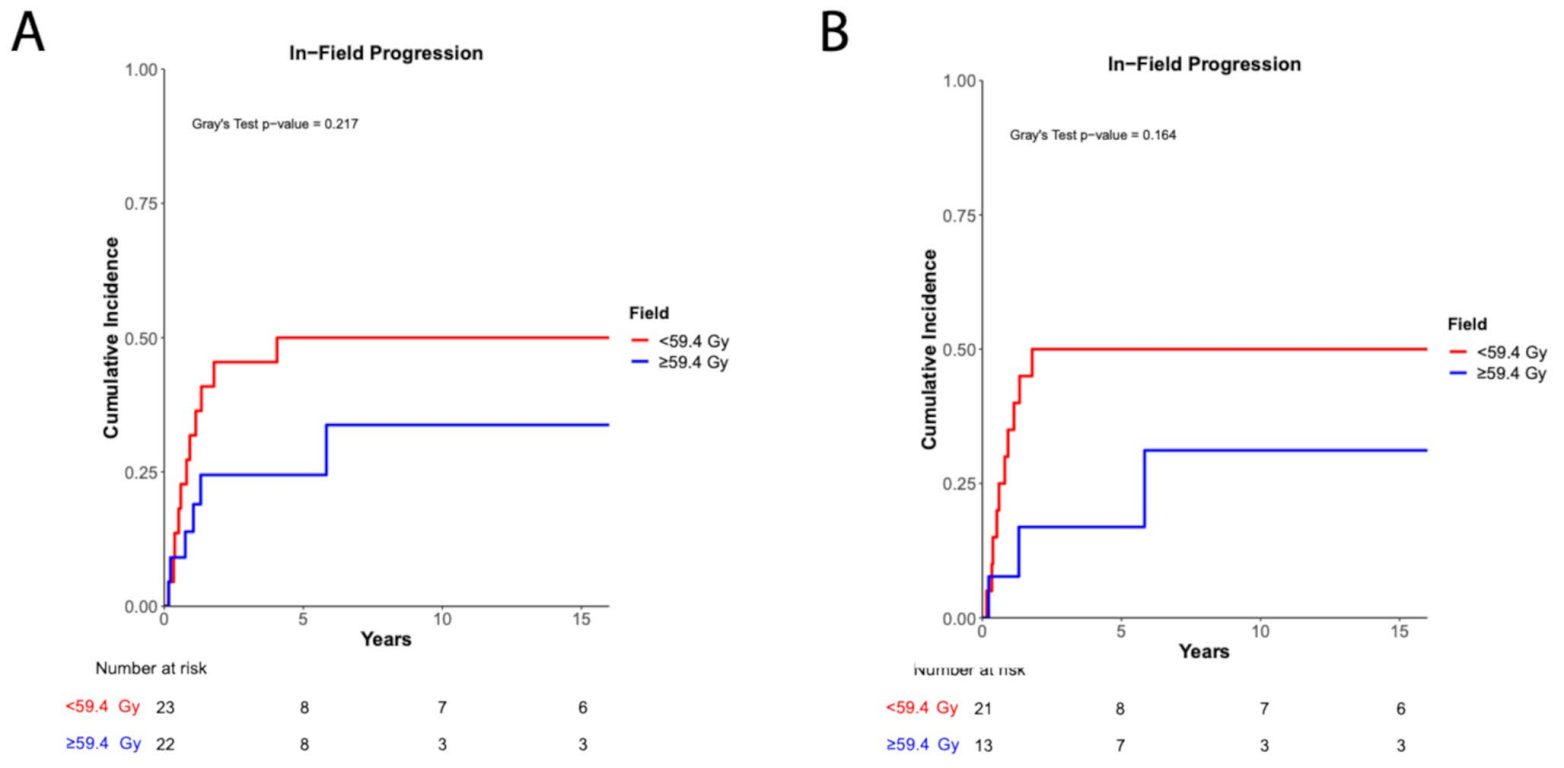
Cumulative incidence of local progression. **(a)** Cumulative incidence of local progression of the entire cohort (*p* = 0.22, Gray’s test). **(b)** Cumulative incidence of local progression of patients with grade 2 ependymoma (*p* = 0.16, Gray’s test)

We did not find a difference in 5-year CILP between the < 59.4 Gy and ≥ 59.4 Gy groups within the entire cohort (50.0% vs. 24.4%, *p* = 0.22, Fig. 2a) or among patients with grade 2 ependymoma (50.0% vs. 16.9%, *p* = 0.16, Fig. 2b).

### Disease progression

We next investigated clinical and treatment characteristics that correlated with disease progression after radiotherapy with death unrelated to disease progression as a competing risk (Table 2). On univariable analysis, age at diagnosis > 4 years was associated with a significantly lower risk of disease progression (subhazard ratio [SHR]: 0.40, 95% confidence interval [CI]: 0.18–0.89, *p* = 0.03). There was a trend towards a higher risk of disease progression in patients who received chemotherapy after radiation (SHR: 2.57, 95% CI: 0.95–6.95, *p* = 0.06). Other characteristics, including resection status, receipt of chemotherapy before radiotherapy, progression before radiotherapy, and radiotherapy dose (Supplemental Figure S1), were not associated with time to disease progression. Of note, the risk of progression was significantly higher in patients who underwent STR up until 5 years after radiation; however, there were multiple late recurrences in patients who underwent GTR that ultimately contributed to a non-significant association between extent of resection and progression (Supplemental Figure S2).

**Table 2:** Univariable analyses for risk of disease progression after radiation with death as a competing risk

	Univariate	
	SHR (95% CI)	p-value
Sex		
Female	Ref.	
Male	1.49 (0.56–3.98)	0.42
Race/Ethnicity		
Non-Hispanic White	Ref.	
Other	0.55 (0.23–1.34)	0.19
Age at diagnosis		
≤4 years	Ref.	
>4 years	0.40 (0.18–0.89)	0.03*
Histological Grade		
Grade 2	Ref.	
Grade 3	1.64 (0.69–3.86)	0.26
Pre-RT chemotherapy		
No	Ref.	
Yes	0.96 (0.37–2.48)	0.93
Disease Progression before RT		
No	Ref.	
Yes	0.98 (0.31–3.10)	0.97
Resection status before RT		
GTR	Ref.	
STR	1.26 (0.44–3.63)	0.67
Number of surgeries before RT		
1	Ref.	
2 or more	1.27 (0.49–3.30)	0.62
Post-RT chemotherapy		
No	Ref.	
Yes	2.57 (0.95–6.95)	0.06
RT dose		
<59.4 Gy	Ref.	
≥59.4 Gy	0.66 (0.30–1.47)	0.31
Days after last surgery to RT		
≤42 days	Ref.	
>42 days	1.38 (0.53–3.57)	0.51

*Indicates significant at *p* < 0.05

### Characteristics and survival of patients with relapsed disease

We also identified 24 patients who had disease progression after radiotherapy. Seven patients were included in a prior study [13] published by our group describing outcomes in patients with relapsed ependymoma. Table 3 summarizes the clinical, upfront, and salvage treatment characteristics of the 24 patients who had relapsed disease. The median time to relapse after radiotherapy was 0.8 years (range: 0.2–9.7). The location of first progression after radiation was metastatic in 3 patients (12.5%) and localized in the supratentorial area, infratentorial area, and spine in 1 (4.2%), 17 (70.8%), and 3 (12.5%) patients, respectively. The location of first progression after radiation was in-field in 17 patients (70.8%). At the time of relapse, 14 (60.9%), 11 (47.8%), and 10 (43.5%) patients received salvage surgery, radiotherapy, and systemic therapy, respectively. Median follow-up time post-relapse was 1.5 years (range: 0.2–7.2). At last follow-up, two (8.3%) patients were alive without evidence of disease, two (8.3%) were alive with disease, 18 (75.0%) died of disease, and 1 (3.7%) died of toxicity related to salvage treatment. We next examined risk factors associated with post-relapse survival using the Cox proportional hazards model (Table 4). Only receipt of salvage systemic therapy remained significantly associated with OS on multivariable analysis (HR = 2.84, 95% CI: 1.05–7.66, *p* = 0.04); there was a trend towards worse OS among patients who underwent STR prior to radiation (HR = 3.77, 95% CI: 0.97–14.72, *p* = 0.06).

**Table 3:** Clinical and treatment characteristics of patients with relapse

	Cohort of patients with relapse (*n* = 24) *n* (%)
Sex	
Female	6 (25.0%)
Male	18 (75.0%)
Age at diagnosis	
≤4 years	15 (62.5%)
>4 years	9 (37.5%)
Histological Grade	
Grade 2	17 (70.8%)
Grade 3	7 (29.2%)
Pre-RT chemotherapy	
No	19 (79.2%)
Yes	5 (20.8%)
Disease Progression before initial RT	
No	21 (87.5%)
Yes	3 (12.5%)
Resection status before initial RT	
GTR	19 (79.2%)
STR	5 (20.8%)
Number of surgeries before initial RT	
1	18 (85.0%)
2 or more	6 (25.0%)
Upfront radiotherapy dose	
<59.4 Gy	14 (58.3%)
≥59.4 Gy	10 (41.7%)
Post-RT chemotherapy	
No	19 (79.2%)
Yes	5 (20.8%)
Time to Relapse after radiotherapy	
≤1 year	14 (58.3%)
>1 year	10 (41.7%)
Location of first progression after RT	
Metastatic	3 (12.5%)
Localized supratentorial	1 (4.2%)
Localized infratentorial	17 (70.8%)
Localized spine	3 (12.5%)
First progression after RT was in-field	
No	7 (29.2%)
Yes	17 (70.8%)
Salvage surgery after first progression	
No	9 (39.1%)
Yes	14 (60.9%)
Salvage radiotherapy after first progression	
No	12 (52.2%)
Yes	11 (47.8%)
Salvage systemic therapy after first progression	
No	13 (56.5%)
Yes	10 (43.5%)

**Table 4:** Univariate and multivariable analyses for time to death after post-radiotherapy relapse

	Univariate		Multivariable	
	HR (95% CI)	p-value	HR (95% CI)	p-value
Sex				
Female	Ref.			
Male	0.70 (0.25–1.98)	0.50		
Age at diagnosis				
≤4 years	Ref.			
>4 years	0.65 (0.25–1.71)	0.38		
Histological Grade				
Grade 2	Ref.			
Grade 3	0.35 (0.10–1.27)	0.11		
Pre-RT chemotherapy				
No	Ref.			
Yes	0.57 (0.16–2.01)	0.38		
Disease Progression before RT				
No	Ref.			
Yes	1.99 (0.56–7.17)	0.29		
Resection status before RT				
GTR	Ref.		Ref.	
STR	3.55 (1.04–12.10)	0.04*	3.77 (0.97–14.72)	0.06
Number of surgeries before RT				
1	Ref.			
2 or more	3.69 (1.26–10.78)	0.02*		
Upfront RT dose				
<59.4 Gy	Ref.			
≥59.4 Gy	0.45 (0.16–1.22)	0.12		
Post-RT chemotherapy				
No	Ref.			
Yes	0.67 (0.19–2.38)	0.54		
Time to Relapse after radiotherapy				
≤1 year	Ref.			
>1 year	0.43 (0.16–1.16)	0.10		
In-field first progression after RT				
No	Ref.			
Yes	1.15 (0.41–3.24)	0.79		
Salvage surgery after first progression				
No	Ref.			
Yes	0.80 (0.29–2.20)	0.67		
Salvage radiotherapy after first progression				
No	Ref.			
Yes	0.37 (0.14–1.01)	0.05		
Salvage systemic therapy after first progression				
No	Ref.		Ref.	
Yes	2.69 (1.01–7.17)	0.049*	2.84 (1.05–7.66)	0.04*
Multimodal therapy after first progression				
No	Ref.			
Yes	0.90 (0.33–2.47)	0.84		

*Indicates significant at *p* < 0.05

### Treatment-associated toxicity

One patient in the < 59.4 Gy cohort who experienced multiple surgical complications developed grade 1 brainstem necrosis five months after definitive radiotherapy. Another patient developed grade 3 brainstem necrosis requiring bevacizumab four months after undergoing re-irradiation with protons (54 Gy in 30 fractions [RBE]). One patient in the ≥ 59.4 Gy cohort died of secondary high-grade glioma 9.5 years after completion of radiation treatment.

## Discussion

We present progression and survival data from a multi-institutional cohort of patients with ependymoma treated with a range of radiotherapy doses. We also assess factors associated with disease progression and examine characteristics of a subgroup of patients with relapse after radiation. While our findings support the use of radiotherapy doses ≥ 59.4 Gy in the management of intracranial ependymoma, they also point to underlying biological factors such as age as important drivers of survival after treatment.

Doses ≥ 59.4 Gy were not associated with better OS or PFS in the cohort as a whole; however, we found a trend towards improved outcomes among patients with grade 2 ependymoma treated with higher doses. While no randomized studies have compared radiation dosing regimens in this patient population, a large, prospective study including 131 patients treated to 59.4 Gy demonstrated excellent seven-year event-free survival and OS rates of 69.1% and 81%, respectively [3]. These findings confirmed the safety and feasibility of dose-escalated radiotherapy in the upfront setting for patients ≥ 18 months old. More recently, a trial conducted by the Associazione Italiana di Ematologia e Oncologia Pediatrica (AEIOP) found higher rates of five-year PFS and OS among patients who received an 8 Gy boost to residual disease compared with historical controls [14], supporting the use of high-dose radiation in a patient subgroup with consistently poor outcomes [2]. Several retrospective studies have since examined the impact of dose on survival with varying results. A National Cancer Center Database analysis of patients > 2 years old who received 54 Gy versus ≥ 59.4 Gy failed to show a difference in OS between dose groups [15]. Of note, the median age of patients in this study was 24, significantly higher than the median ages of 2.6 and 9.1 years in the < 59.4 Gy and ≥ 59.4 Gy groups in our cohort. Adolescents and young adults tend to have better outcomes compared with younger children [16] and may therefore benefit less from dose-escalated treatment. This is consistent with results from Ager et al., who found that doses > 54 Gy correlated with improved OS in patients ages 2 to 18 but not in those over 18 [17].

Within the last decade, molecular profiling has led to the discovery of nine distinct ependymoma subgroups [18] that have since replaced classical histopathologic grading in the 2021 WHO classification scheme [19]. These subtypes correlate closely with features such as age of presentation and disease location (i.e., supratentorial versus infratentorial). For example, both the PFA and PFB subtypes are characterized by posterior fossa tumors; however, PFA ependymomas occur primarily in infants and young children while PFB tumors are more often found in adolescents and young adults [20]. Importantly, the PFA subtype has been associated with worse outcomes [18]. This may explain our finding of shorter PFS among patients ≤ 4 years old at diagnosis, although we unfortunately did not have molecular subtyping available for patients in this cohort. Of note, considerable heterogeneity exists within each PF subtype [21, 22] and the prognostic implications of these molecular features remain controversial, with ACNS0121 [2] failing to detect a difference in PFS, OS, or failure pattern between PFA and PFB tumors. Larger studies of more modern ependymoma cohorts with molecular subtyping are needed to further understand the relationship between age and survival.

Notably, STR, which has consistently been linked to poor oncologic outcomes and survival, did not portend an increased risk of progression in this cohort. While the risk of progression up to five years after radiation was clearly higher among patients who underwent STR (Supplemental Figure S1), there were multiple late recurrences in the GTR group that ultimately obscured any early PFS advantage in these patients. The relative infrequency of patients with STR in this study (20.0%, versus 80.0% with GTR) likely also contributed to a non-significant difference in PFS between these groups. Another possible explanation for this finding may be related to underlying imbalances in the distribution of ependymoma subtypes between patients who underwent GTR versus STR. Indeed, these subgroups enhance patient risk stratification compared with other traditional clinical risk factors such as extent of resection [23] and tumor location [20]. Further investigation into how these novel molecular subgroups will influence classic treatment paradigms is needed.

We also describe the clinical features, management, and outcomes among a subset of 24 patients who ultimately relapsed after upfront radiotherapy. The median time to relapse was 0.8 years, consistent with prior studies of recurrent ependymoma [13]. Relapse times among patients in our cohort ranged from 0.2 to 9.7 years, reflecting the potential for a long latency period between initial treatment and disease recurrence that is characteristic of ependymoma [10]. Consistent with prior literature [5], most patients had progression of disease within the original radiation field. Management upon disease recurrence varied. As shown in prior studies [13], salvage surgery (61% of patients) and reirradiation (48%) were the most commonly used treatment modalities at progression. Optimal management at the time of relapse is unknown. Prior studies have shown favorable outcomes among patients who undergo salvage surgery, specifically among those who achieve a GTR [10, 24]. Re-irradiation has also demonstrated efficacy upon recurrence, with three-year OS rates ranging from 48–81% [25–28]. Various radiation modalities have been explored in the relapse setting, including conventionally or hypofractionated focal radiation [24], stereotactic radiosurgery [29], and craniospinal irradiation (CSI) [30, 31]. The role of salvage chemotherapy remains controversial. A wide range of regimens are currently used [24], and it is typically recommended when surgery and radiotherapy are not feasible [32]. Indeed, patients in this cohort and others [10, 24, 33] who received salvage systemic therapy had worse survival, likely due to selection bias for patients unable to get local therapy or those with more widespread disease. The lack of survival advantage seen with surgery, radiation, or chemotherapy in this study reflects the complexity of management of recurrent ependymoma. More robust exploration of optimal treatment strategies at the time of relapse are needed, particularly given that about one-third of patients ultimately recur [34].

Rates of brainstem necrosis, a rare but devastating potential complication of ependymoma treatment, were low in this cohort. This is consistent with prior studies examining the risk of brainstem necrosis after photon radiotherapy to the posterior fossa [35, 36]. One patient developed grade 3 brainstem necrosis after undergoing reirradiation with protons, both of which have been identified as risk factors for brainstem toxicity [37, 38]. Fortunately, modified consensus treatment planning guidelines and revised proton-specific brainstem constraints continue to improve the safety profile of posterior fossa radiation [36].

This study has several limitations, many of which are related to its retrospective nature. We were unable to avoid selection bias towards treating patients with more aggressive disease with higher radiation doses; patients with grade 3 ependymoma and those who received post-radiation chemotherapy were more likely to be treated to ≥ 59.4 Gy. Nevertheless, we also specifically examined the association between radiation dose and survival in patients with grade 2 disease, where there was a trend towards improved OS and PFS with higher radiation doses. Our sample size was relatively small; therefore, comparisons of outcomes between various patient subgroups may have been underpowered. In addition, given the small sample size, we were unable to perform other types of analyses, including propensity score matching. Notably, few patients underwent STR, which may have contributed to our lack of finding a link between extent of resection and risk of progression. Nevertheless, we had long follow-up from patients treated at multiple institutions, including 24 patients with relapsed disease. There was no central review of baseline pathology and imaging, which may have altered the baseline characteristics of patients in our cohort, including assigned histologic grade [39]. Finally, we did not have information on molecular tumor subtypes for these patients and were therefore unable to determine whether subgroups were balanced between the < 59.4 Gy and ≥ 59.4 Gy cohorts. Notably, patients receiving < 59.4 Gy were significantly younger; therefore, it is possible that these patients demonstrated earlier progression due to underlying biological differences in their disease (i.e., higher likelihood of PFA subtype) rather than suboptimal radiation dosing. This may also explain why traditional risk factors such as upfront extent of resection did not appear to influence survival in this study. It is unclear if our findings would have changed had these subgroups been considered.

In conclusion, we present the characteristics, management, and outcomes from a multi-institutional cohort of patients with intracranial ependymoma treated with radiotherapy. Our findings suggest that higher radiation doses may improve outcomes in patients with grade 2 disease and confirm the poor prognostic significance of younger age. Further research into the management of relapsed ependymoma is needed. Optimal radiation dosing remains unknown in the era of molecular subtyping. Prospective trials that describe outcomes among molecular subgroups such as COG ACNS0121 [2] and ACNS0831 [11] may provide better insight into which patients benefit most from higher doses.

## Electronic supplementary material

Below is the link to the electronic supplementary material.

Supplementary Material 1

Supplementary Material 2

## Data Availability

The datasets generated during and/or analyzed during the currenty study are available from the corresponding author on reasonable request.
