# Supplementary figures and images for: The association between postoperative photon radiotherapy dose and disease control and salvage treatment in pediatric and adolescent ependymoma: a multi-institutional investigation
Source: J Neurooncol. 2025 Feb 25;173(1):167–77. doi: 10.1007/s11060-025-04975-5 (PMC12040991; doi:10.1007/s11060-025-04975-5)

A

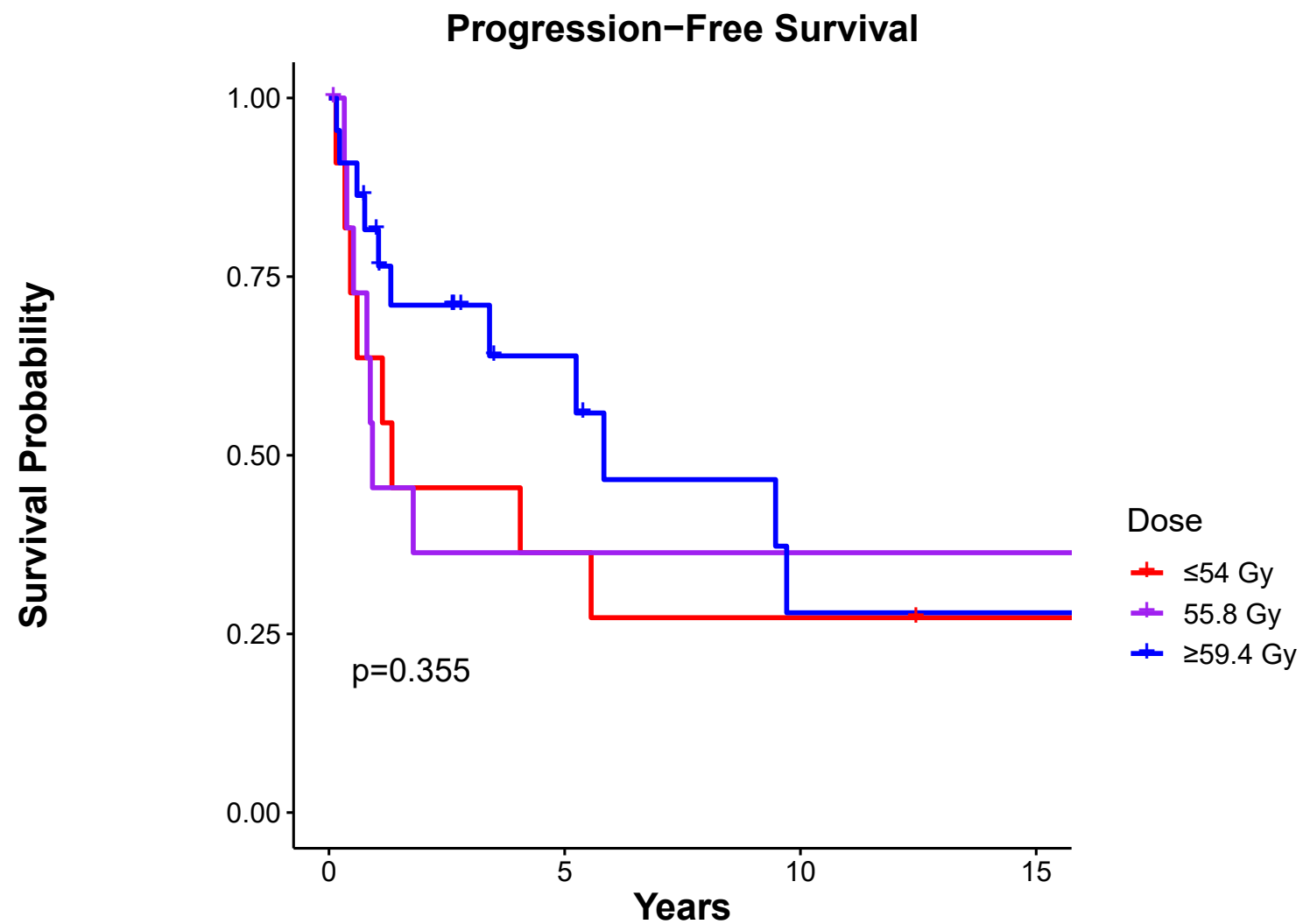

| Number at risk |    |   |    |    |
|----------------|----|---|----|----|
|                | 0  | 5 | 10 | 15 |
| ≤54 Gy         | 11 | 4 | 3  | 2  |
| 55.8 Gy        | 12 | 4 | 4  | 4  |
| ≥59.4 Gy       | 22 | 8 | 3  | 3  |

B

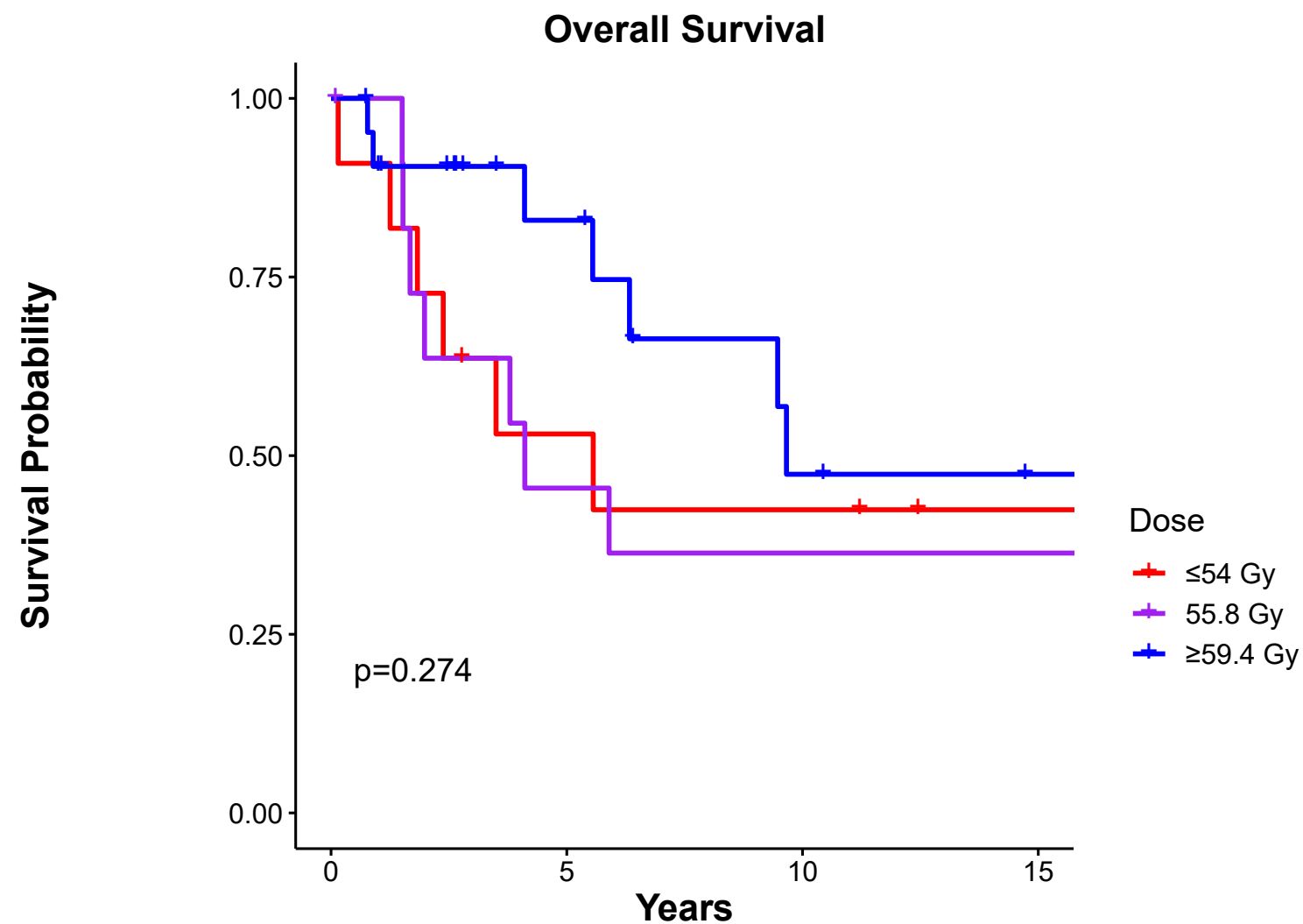

| Number at risk |    |    |    |    |
|----------------|----|----|----|----|
|                | 0  | 5  | 10 | 15 |
| ≤54 Gy         | 11 | 5  | 4  | 2  |
| 55.8 Gy        | 12 | 5  | 4  | 4  |
| ≥59.4 Gy       | 22 | 11 | 5  | 3  |

Supplement: Supplementary file 1 — Supplementary Material 1 [file 11060_2025_4975_MOESM1_ESM.pdf]

# Progression-Free Survival

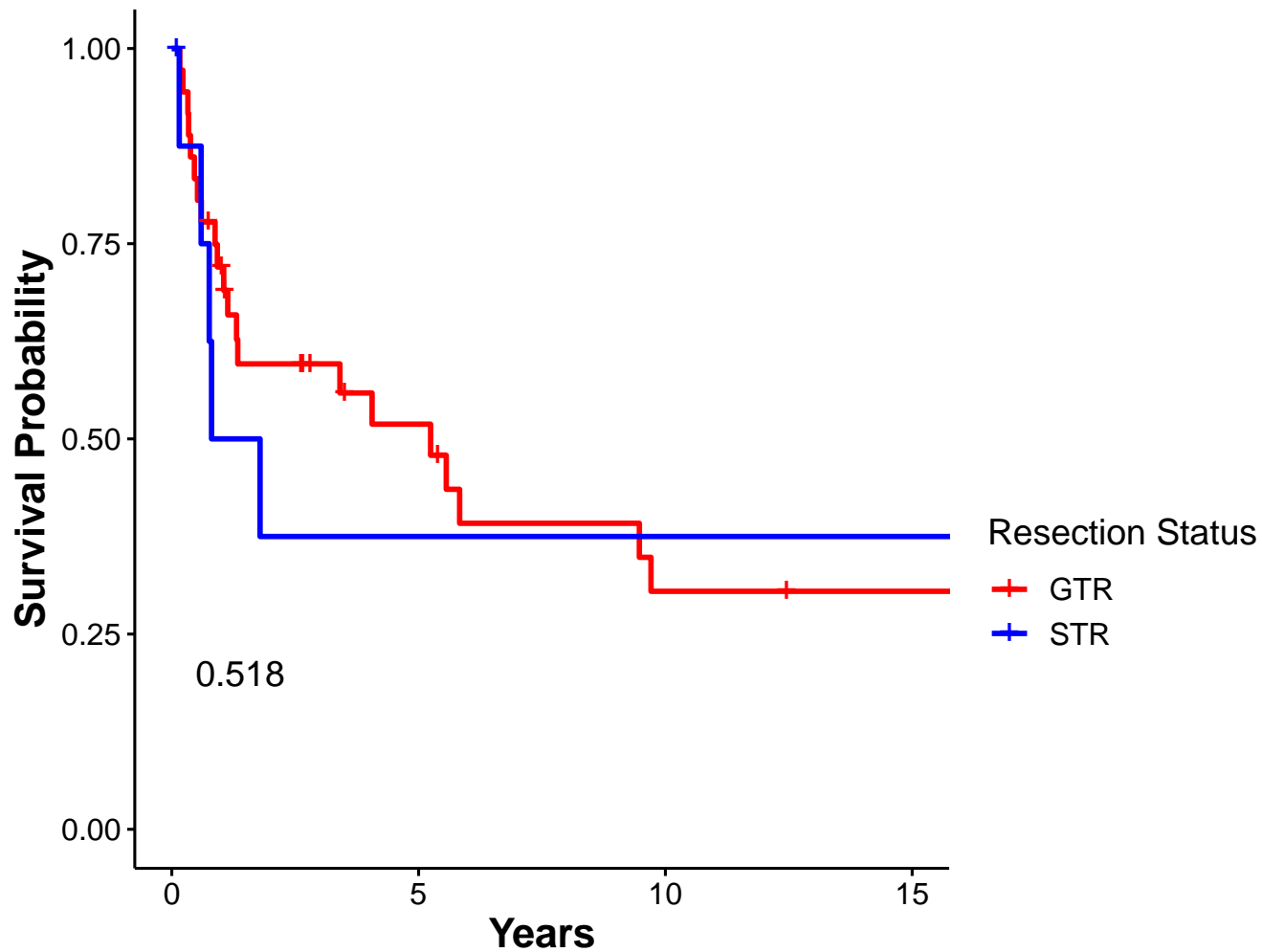

Number at risk

|     |    |    |   |   |
|-----|----|----|---|---|
| GTR | 36 | 13 | 7 | 6 |
| STR | 9  | 3  | 3 | 3 |

Supplement: Supplementary file 2 — Supplementary Material 2 [file 11060_2025_4975_MOESM2_ESM.pdf]
